# Levels of circulating endothelial progenitor cells inversely correlate with manic and positive symptom severity in patients with bipolar disorder

**DOI:** 10.1002/brb3.2570

**Published:** 2022-04-28

**Authors:** Ying‐Jay Liou, Mu‐Hong Chen, Ju‐Wei Hsu, Kai‐Lin Huang, Po‐Hsun Huang, Ya‐Mei Bai

**Affiliations:** ^1^ Department of Psychiatry Taipei Veterans General Hospital Taipei Taiwan; ^2^ Department of Psychiatry, School of Medicine National Yang Ming Chiao Tung University Taipei Taiwan; ^3^ Institute of Brain Science National Yang Ming Chiao Tung University Taipei Taiwan; ^4^ Division of Cardiology, Department of Medicine Taipei Veterans General Hospital Taipei Taiwan; ^5^ Department of Critical Care Medicine Taipei Veterans General Hospital Taipei Taiwan; ^6^ Cardiovascular Research Center National Yang Ming Chiao Tung University Taipei Taiwan

**Keywords:** biomarkers, bipolar disorder, cardiovascular diseases, circulating endothelial progenitor cells

## Abstract

**Objectives:**

Patients with bipolar disorder (BPD) are at high risk of cardiovascular diseases (CVDs) that are attributed to endothelial dysfunction. Circulating endothelial progenitor cells (cEPCs) are proposed as indicators of endothelial dysfunction. This study examined the relationship of cEPC numbers with BPD diagnosis and its clinical symptoms in patients with BPD.

**Methods:**

We recruited 48 patients with BPD and 50 healthy controls (HCs). All the patients had scores of <13 on the Young Mania Rating Scale (YMRS). In addition to the YMRS and Clinical Global Impression for BPD (CGI‐BP), bipolar patients were assessed using relevant measurements for their depression, anxiety, general psychopathology, cognitive dysfunction and deficit, somatic symptoms, quality of life, and level of disability. cEPC counts were measured using flow cytometry.

**Results:**

The numbers of immature and mature cEPCs in the bipolar patients did not significantly differ from those in the HCs. After correction for multiple comparisons and controlling for body mass index and smokers, the number of immature cEPCs was observed to be inversely correlated with CGI‐BP (corrected *p* [*p_corr_*] = .00018) and positive scores in the positive and negative syndrome scale (PANSS‐P; *p_corr_* = .0049). The number of mature cEPCs was inversely correlated with YMRS (*p_corr_* = .014), CGI‐BP (*p_corr_* = .00022), and PANSS‐P (*p_corr_* = .0049) scores. In multivariate stepwise analysis, numbers of both types of cEPCs were associated with CGI‐BP.

**Conclusions:**

cEPC levels, an indicator of endothelial dysfunction and risk of CVDs, may be associated manic and positive symptom severities in patients with BPD.

## 1 INTRODUCTION

Bipolar disorder (BPD) is chronic, lifelong, recurrent, complex, and multifactorial psychiatric disorder of mood that is characterized by manic, hypomanic, or depressive episodes. Evidence suggests that patients with BPD are more susceptible to CVDs, including ischemic heart disease, congestive heart failure, and cerebrovascular disorders (Carney & Jones, 2006). In a 3‐year longitudinal study, individuals with BPD showed a higher incidence of CVDs and were more than twice as likely to have received a CVD diagnosis compared with patients with major depressive disorder and controls (Goldstein et al., 2015). CVD onset also occurred at a younger age in patients with BPD compared with people with major depressive disorder and controls (Goldstein et al., 2015). Although the higher rates of CVD comorbidity in patients with BPD may be attributed to the use of mood stabilizers and antipsychotic drugs for the treatment of the disease, some researchers have rejected this hypothesis, demonstrating that such medications are not associated with higher CVD mortality or risk of myocardial infarction or cerebrovascular disorder (Rotella et al., 2020), and no definitive evidence suggests that these medications independently increase CVD risk (Goldstein et al., 2020). These findings suggest that other pathophysiological mechanisms (e.g., lifestyle‐related behaviors and cardiovascular factors) underlie the association between BPD and CVD susceptibility.

The earliest stage of atherogenesis and CVD is endothelial dysfunction, which suggests a link between the cardiovascular risk factors and atherosclerosis‐related disease as well as long‐term CVD outcomes (Amarasekera et al., 2021). The vascular endothelium is an organ with active paracrine, endocrine, and autocrine functions that are crucial for the regulation of vascular tone, maintenance of vascular homeostasis, and mediation of inflammation (Little et al., 2021). Endothelial dysfunction impairs the endothelium‐dependent vasodilation and activation of proinflammatory, proliferative, and procoagulant activities. It thus increases an individual's susceptibility to atherosclerotic diseases and unfavorable cardiovascular outcomes (Little et al., 2021).

Recent studies suggested that endothelial dysfunction and injury to the vascular wall are repaired by circulating endothelial progenitor cells (cEPCs) derived from the bone marrow (Chopra et al., 2018). These cells express numerous hematopoietic stem cell specific (e.g., CD34 and CD133), progenitor cell specific (e.g., CD133), and endothelial cell specific (e.g., kinase insert domain receptor [KDR]) surface markers, and they can be classified as immature (or early; positive for CD34, CD133, and KDR) and mature (or late; CD34 and KDR) cEPCs (Chopra et al., 2018). In response to tissue ischemia caused by endothelial dysfunction and injury, cEPCs differentiate from hematopoietic stem cells derived from the bone marrow. They then mobilize into the peripheral blood, migrate to vascular damage sites, integrate into the endothelial monolayer, and subsequently promote vascular repair and angiogenesis through paracrine signaling to neighboring cells and transdifferentiating to mature endothelial cells (Chopra et al., 2018). The cEPC count is correlated with clinical surrogates of endothelial dysfunction (e.g., flow‐mediated brachial artery reactivity and carotid intima media thickness) and is associated with combined Framingham risk factor scores (Chopra et al., 2018; Hill et al., 2003). Therefore, cEPCs have been proposed as direct indicators of endothelial function (Chopra et al., 2018).

Despite increasing evidence demonstrating the involvement of cEPCs in CVDs and the association of BPD with the increased risk of CVDs, the relationship between cEPCs and BPD and its related clinical manifestations has not yet been thoroughly studied. Ferensztajn‐Rochowiak et al. (2017) discovered that the cEPC numbers of patients with BPD (some of whom were treated with lithium) did not differ significantly from those of controls. However, that study did not investigate the association of cEPC numbers with the clinical symptoms of BPD (Ferensztajn‐Rochowiak et al., 2017). Therefore, the present study investigated whether the numbers of cEPCs are associated with either BPD or its clinical presentations, including manic symptom severity, cognitive dysfunction, somatic symptoms, subjective disability in key functional domains, and suboptimal quality of life.

## 2 METHODS

### 2.1 Patients and participants

This study was conducted at Taipei Veterans General Hospital (Taipei, Taiwan). Board‐certificated senior psychiatrists interviewed all the participants using the Mini International Neuropsychiatric Interview (MINI), clinical observation, and medical charts to diagnose psychiatric disorders. The diagnosis of BPD was made based on the criteria for BPD specified in the Diagnostic and Statistical Manual of Mental Disorders, Fourth Edition, Text Revision (DSM‐IV‐TR). The exclusion criteria were any DSM‐IV‐TR diagnosis (e.g., schizophrenia, other psychotic disorders, intellectual disability, substance use disorder, or psychiatric disorder due to general medical condition or substance use), unpredictable physical illness, pregnancy, and breastfeeding. Age‐ and gender‐matched participants without any psychiatric disorder were included in the healthy control (HC) group. The participants’ ages ranged from 20 to 65 years. This study was approved by the Institutional Review Board of Taipei Veterans General Hospital and was conducted in accordance with the Declaration of Helsinki. Written informed consent was obtained from the participants prior to their study involvement.

### 2.2 Clinical assessments

This study assessed the severity of manic symptoms by using the YMRS and Clinical Global Impressions Bipolar (CGI‐BP) scale (Spearing et al., 1997). The YMRS has 11 items and is one of the most widely employed scales for evaluating manic symptoms according to a patient's subjective condition. Additional information derived from clinical observation was elicited during the course of the interview (Young et al., 1978).

The severity of current depressive symptoms was evaluated using the 17‐item Hamilton Depression Rating Scale (HAMD‐17) and Montgomery–Åsberg Depression Rating Scale (MADRS). Both scales are commonly applied in psychiatric research and clinical practice for assessing depression level. However, the HAMD‐17 does not evaluate oversleeping, overeating, and concentration difficulties, and the MADRS lacks assessment of psychomotor changes, reduced interest in daily activities, or guilt (Carmody et al., 2006). Because neither the HAMD‐17 nor MADRS can be used to evaluate all of the core criterion symptoms of a major depressive episode, both scales were used to fully assess depressive symptom severity in the patients with BPD in this study.

This study also used the positive and negative syndrome scale (PANSS) to assess the level of general BPD psychopathology (Kay et al., 1987). PANSS was originally designed to evaluate schizophrenia‐related psychotic symptoms, and it comprises three components: positive symptoms (PANSS‐P), negative symptoms (PANSS‐N), and general symptoms (PANSS‐G) (Kay et al., 1987). The scale has also been widely applied to assess affective and psychotic symptoms in patients with BPD (Anderson et al., 2017).

In the present study, the Digit Symbol Substitution Test (DSST) was employed to assess the participants' objective neurocognitive function. The DSST measures the psychomotor speed of the performance in tasks requiring visual perception, spatial decision making, and motor skills. The DSST is a valid and sensitive assessment of various cognitive domains, including executive function, attention, psychomotor speed, and working memory (Jaeger, 2018). In addition, the participants subjectively assessed their cognitive deficits by completing the Perceived Deficits Questionnaire for Depression (PDQD) (Lam et al., 2018). The PDQD assesses the impact of cognitive dysfunction on an individual's ability to perform activities of daily living based on their self‐reported experiences in the context of BPD symptoms.

The participants also completed two other self‐administered questionnaires: the Depressive and Somatic Symptom Scale (DSSS) and the 12‐item Short‐Form Health Survey (SF‐12). The advantage of the DSSS is that it simultaneously assesses somatic and depressive symptoms, overcoming the deficiencies of other depression scales that assess few somatic elements (Hung et al., 2006). SF‐12 comprises summaries of physical components (SF12‐PCS) and mental components (SF12‐MCS) and evaluates an individual's overall health‐related quality of life (Ware et al., 1996).

Finally, this study also used the Sheehan Disability Scale (SDS) to assess patients' functional impairment in daily life. The SDS is a three‐item self‐reported tool for evaluating functional disability in work, school, social, and family life contexts (Sheehan & Sheehan, 2008).

### 2.3 Measurement of biochemical parameters and cEPC counts

Blood samples were drawn after 12‐h overnight fasting. Plasma biochemical parameters, such as alanine aminotransferase (ALT), aspartate aminotransferase (AST), creatinine (CREAT), fasting blood glucose (FBS), triglyceride (TG), cholesterol (CHOL), high‐density lipoprotein cholesterol (HDL), and uric acid (UA) levels, were determined using standard laboratory procedures. For the measurement of cEPC counts, a 1.0‐ml sample of peripheral blood was obtained from each participant. The cEPC count was measured through flow cytometry by researchers blinded to the patients’ clinical information. The blood samples were subsequently incubated with allophycocyanin (APC)‐conjugated monoclonal antibodies against human KDR (R&D, Minneapolis, MN, USA), phycoerythrin (PE)‐conjugated monoclonal antibodies against human CD133 (Miltenyi Biotec, Germany), and fluorescein isothiocyanate (FITC)‐conjugated monoclonal antibodies against human CD34 (BD Biosciences Pharmingen, San Diego, CA, USA) in the dark for 30 min. Each analysis was based on 150,000 acquired events and was as reliable as 500,000 events (intraclass correlation coefficient > 0.95). When viability markers were included, the viability of cEPCs reached 96.3%. The interindividual variability of two samples obtained from 10 patients was strongly correlated (*r *= .90, *p *< .001). The intraindividual variability of immature (CD34+KDR+CD133+) and mature (CD34+KDR+) cEPCs over time was evaluated based on two measurements conducted 1 year apart in 21 patients, and the resulting intraclass correlation coefficients were 0.69, 0.75, and 0.78, respectively. Cell counts are expressed as cEPCs per 10^5^ mononuclear cells (Liou et al., 2021).

### 2.4 Statistical analyses

Statistical analyses were performed using SPSS (version 21; SPSS Inc., Chicago, IL, USA). The distribution of categorical variables across groups was compared using the chi‐square test (and the Fisher exact test if necessary). The group differences in continuous variables were determined using the independent *t* test or the Mann–Whitney *U* test if the variable deviated from the assumption of a normal distribution. The normality of continuous variables was examined with the Shapiro–Wilk test. The direction and strength of the correlation between cEPC counts and clinical measurements were examined with Kendall's tau‐b partial correlation in three models by using JASP 0.16 (https://jasp‐stats.org/download/). Model 1 controlled for the effect of body mass index (BMI) and smokers. Model 2 additionally controlled for systolic blood pressure (SBP), diastolic blood pressure (DBP), FBS, and HDL in addition to controlling for BMI and smokers. Model 3 additionally controlled for the use of antipsychotic drugs and mood stabilizers in addition to controlling for BMI and smokers. Models 2 and 3 are the parallel sensitivity analyses. Multivariate stepwise linear regression was used to identify the most predictive clinical symptom associated with cEPC counts with adjustment for potential confounding factors (i.e., BMI, SBP, DBP, smokers, FBS, and HDL). The criterion for entry an independent variable was set as if a *p*‐value less than .05 and for removal as greater than .1. The statistical significance level for all univariate analyses was set at a corrected *p‐*value of <.05 after multiple comparison adjustment with the Benjamini–Hochberg procedure (https://tools.carbocation.com/FDR), and the threshold for statistical significance in the multivariate stepwise linear regression was set as *p* <.05.

## 3 RESULTS

### 3.1 cEPC numbers in patients with BPD and in HCs

In this study, 48 patients with BPD (bipolar I = 24, bipolar II = 24) and 50 HCs were recruited. The BPD patients were treated with the following psychotropic drugs: antipsychotic drugs (risperidone = 3, olanzapine = 1; quetiapine = 10, ziprasidone = 3, lurasidone = 18, aripiprazole = 3, amisulpride = 1, and sulpiride = 1), mood stabilizers (lithium = 5; valproic acid = 6), and antidepressants (fluoxetine = 2; excitalopram = 2; sertraline = 4; venlafaxine = 4; duloxetine = 1; bupropion = 4; agomelatine = 2; mertazapine = 1; lamotrigine = 6). Some of the patients had also received diagnoses of hypertension (*N* = 2), heart disease (*N* = 1), diabetes mellitus (DM; *N* = 5), and hyperlipidemia (*N* = 2). The total YMRS score of all patients with BPD was less than 13. The mean (range, SD) scores of YMRS, HAMD‐17, and MADRS in the BPD patients were 2.4 (0–11, 2.8), 10.9 (0–30, 6.4), and 15.1 (0–36, 9.1), respectively. Table 1 lists the demographic and clinical data, laboratory data, and cEPC counts of the BPD and HC groups. The between‐group differences in gender distribution and in the median age, height, heart rate, and ALT, AST, UA, CHOL, HDL, and low‐density lipoprotein (LDL) cholesterol levels were all nonsignificant. However, the BPD group included more smokers than the HC group. The BPD group also exhibited significantly higher median body weight (BW), BMI, SBP, DBP, and TG level but a lower median FBS level than the HC group (Table 1; Mann–Whitney *U* test, all *p* <.05). No significant difference was observed in mature and immature cEPC counts between the patients with BPD and the HCs (Table 1; Mann–Whitney *U* test, all *p* >.05).

**TABLE 1:** Comparison of demographic parameters, clinical measurements, and cEPC counts between patients with bipolar disorder (BPD) and healthy controls (HC).^a^

	BPD (*N* = 48)	HC (*N* = 50)	*p* (χ^2^ or MWU)^b^
Gender, male/female	20/28	21/29	.973
Smoker, yes/no	15/33	1/49	***<.001***
Age, year	26 (15)	27(11)	.850
Illness duration, year	6.5 (12.5)		
Treatment duration, year	0.83 (7.9)		
Height, cm	165.7 (10.4)	164.0 (15.0)	.398
BW, kg	65.3 (23.8)	60.0 (17.8)	***.029***
BMI	24.8 (8.6)	22.3 (3.3)	***.037***
SBP, mmHg	116.0 (13.0)	112.0 (15.0)	***.038***
DBP, mmHg	71.0 (11.0)	65.0 (11.0)	***.007***
Heart rate	75.0 (14.0)	71.5 (13.0)	.064
ALT, U/L	17.0 (17.0)	15.5 (9.0)	.291
AST, U/L	18.0 (7.0)	18.5 (6.0)	.935
CREAT, mg/dl	0.8 (0.2)	0.75 (0.14)	.871
UA, mg/dl	5.3 (1.8)	5.6 (1.8)	.446
FBS, mg/dl	81.5 (15.0)	85.5 (9.0)	***.024***
CHOL, mg/dl	183.5 (42.0)	188.5 (54)	.823
TG, mg/dl	96.0 (69.0)	70.5 (38.0)	.052
HDL, mg/dl	50.5 (20.0)	56.0 (16.0)	***.037***
LDL, mg/dl	109.0 (42.1)	110.1 (41.4)	.725
Mature cEPCs, counts	16.0 (17.0)	13.0 (16.0)	.240
Immature cEPCs, counts	11.0 (12.0)	11.0 (14.0)	.834

Abbreviations: ALT: alanine aminotransferase; AST: aspartate aminotransferase; BMI: body mass index; BW: body weight; CREAT: creatinine; cEPC: circulating endothelial progenitor cells; CHOL: cholesterol; DBP: diastolic blood pressure; FBS: fasting blood sugar; HDL: high‐density lipoprotein cholesterol; LDL: low‐density lipoprotein; MWU: Mann‐Whitney U test; TG: triglyceride; SBP: systolic blood pressure; UA: uric acid.

^a^ Continuous variables were presented as their medians and interquartile ranges (IQR).

^b^ Bold italicized values represent the *p*‐value less than .05.

### 3.2 Strength (degree) and direction of correlation between cEPC count and BPD‐related clinical indicators

Table 2 presents the correlation coefficients (τ_b_) between the cEPC counts and clinical measurements related to BPD in three analytic models with Kendall tau‐b partial correlations. Both immature and mature cEPC counts were correlated inversely with YMRS in Model 1 (immature: *p* = .011; mature: *p* = .002), Model 2 (immature: *p* = .014; mature: *p* = .0029), and also in Model 3 (immature: *p* = .008; mature: *p* = .0012). The counts of both types of cEPCs were also correlated inversely with CGI‐BP in Model 1 (immature: *p* = .0000082; mature: *p* = .0000024), Model 2 (immature: *p* = .000012; mature: *p* = .0000029), and Model 3 (immature: *p* = .000016; mature: *p* = .0000032). The score of PANSS‐P were found to correlate negatively with both types of cEPCs in Model 1 (immature: *p* = .0006; mature: *p* = .00049), Model 2 (immature: *p* = .00072; mature: *p* = .0005), and Model 3 (immature: *p* = .00025; mature: *p* = .00015). Despite the correlation noted between immature cEPCs and YMRS scores, inverse correlations of the mature cEPCs count with the YMRS scores, immature and mature cEPC counts with the CGI‐BP scores, and immature and mature cEPC counts with the PANSS‐P scores in the three partial correlation models remained statistically significant after correction for multiple comparisons (all corrected *p* < .05).

**TABLE 2:** Kendall Tau‐b partial correlation analysis for circulating endothelial progenitor cell numbers and clinical symptomatology level in patients with bipolar disorder.^a^

	Immature cEPCs			Mature cEPCs		
	Model 1^b^	Model 2^c^	Model 3^d^	Model 1^b^	Model 2^c^	Model 3^d^
YMRS	−0.26*	−0.26*	−0.28**	−0.31**	−0.31**	−0.34**
CGI‐BP	−0.46***	−0.47***	−0.45***	−0.48***	−0.50***	−0.49***
HAMD	−0.11	−0.14	−0.12	−0.07	−0.10	−0.09
MADRS	−0.16	−0.17	−0.19	−0.16	−0.18	−0.15
HAMA	−0.19	−0.21	−0.16	−0.17	−0.19	−0.15
PANSS‐T	−0.20*	−0.19	−0.19	−0.21*	−0.19	−0.20
PANSS‐P	−0.35***	−0.36***	−0.38***	−0.36***	−0.37***	−0.40***
PANSS‐N	−0.17	−0.13	−0.14	−0.14	−0.10	−0.11
PANSS‐G	−0.14	−0.16	−0.15	−0.16	−0.17	−0.17
DSSS	−0.00	−0.18	−0.12	−0.10	−0.15	−0.09
DSST	−0.15	−0.04	−0.03	0.00	0.03	0.02
PDQD	−0.15	−0.16	−0.16	−0.18	−0.18	−0.19
PCS	−0.11	−0.14	−0.08	−0.08	−0.12	−0.05
MCS	0.18	−0.11	−0.03	−0.16	0.12	−0.17
SDS	−0.17	−0.10	−0.02	−0.11	0.04	−0.05

Abbreviations: CGI‐BP: Clinical Global Impression—Bipolar Version—Severity of Illness; DSSS: Depressive and Somatic Symptom Scale; DSST: Digit Symbol Substitution Test; HAMA: Hamilton Anxiety Rating Scale; HAMD: 17‐item Hamilton Depression Rating Scale; MADRS: Montgomery–Åsberg Depression Rating Scale; MCS: Mental Component Summary of the 12‐Item Short Form Health Survey; PCS: Physical Component Summary of the 12‐Item Short Form Health Survey; PANSS‐G: general scores in PANSS; PANSS‐P: positive scores in PANSS; PANSS‐N: negative scores in PANSS; PANSS‐T: total scores in Positive and Negative Syndrome Scale (PANSS); PDQD: Perceived Deficits Questionnaire‐Depression; SDS: Sheehan Disability Scale; YMRS: Young Mania Rating Scale.

^a^ The number in each cell corresponds to the Kendall's tau‐b correlation coefficient between variables. *p < .05, **p < .01, ***p < .001.

^b^ Model 1: controlling for BMI and smokers.

^c^ Model 2: Model 1 additionally controlling for SBP, DBP, FBS, and HDL.

^d^ Model 3: Model 1 additionally controlling for the use of antipsychotic drugs and mood stabilizers.

### 3.3 Effect of symptom levels of BPD on cEPC counts

In this study, linear regression was used to determine the relationship between the cEPC count and clinical information with simultaneous adjustment for potential confounding factors (e.g., BMI, SBP, DBP, FBS, CHOL, TG, and smoking). In the analyses, cEPC counts were the dependent variables, and scores of the clinical measures were the independent variables. Table 3 presents the results of univariate and multivariate analyses. The univariate analysis indicated that cholesterol level (*p* = .044), YMRS score (*p* = .030), CGI‐BP score (*p* = .002), and PANSS‐Total score (PANSS‐T; *p* = .041) were significantly associated with the immature cEPC count. Among these results, the association between CGI‐BP scores and the immature cEPC count remained statistically significant after correction for multiple testing (corrected *p* = .04). The mature cEPC count was significantly associated with being a smoker (*p* = .013) as well as YMRS (*p* = .021), CGI‐BP (*p* = .002), PANSS‐T (*p* = .016), and PANSS‐G (*p* = .044) scores in the univariate analysis. Among these results, the association between the mature cEPC count and CGI‐BP score remained statistically significant after correction for multiple testing (corrected *p* = .044). In the multivariate stepwise linear analysis, the CGI‐BP score was significantly associated with both immature and mature cEPC numbers (*p* = .003 and .006, respectively).

**TABLE 3:** Linear regression for the association of cEPC counts with clinical assessments related to BPD

	Immature cEPC					Mature cEPC				
	Univariate			Multivariate		Univariate			Multivariate	
	β (95% CI)	*p* ^a^	Corrected *p* ^b^	β (95% CI)	*p* ^a^	β (95% CI)	*p* ^a^	Corrected *p* ^b^	β (95% CI)	*p* ^a^
BMI	−0.93(−0.56, 0.37)	.691				−0.02(−0.62, 0.58)	0.938			
SBP	0.046(−0.14, 0.23)	.622				0.10(−0.14, 0.33)	0.827			
DBP	0.06(−0.19, 0.31)	.624				0.16(−0.16, 0.48)	.318			
FBS	.03(−0.09, 0.15)	0.605				0.06(−0.09,0.21)	.437			
HDL	0.07(−0.11‐0.24)	.457				0.08(−0.14‐0.31)	.472			
Smoker	4.82(−1.05, 10.70)	.106				9.5(2.06, 16.9)	***.013***			
YMRS	−1.34(−2.62, −0.14)	***.030***				−2.03(−3.74, −0.32)	***.021***			
CGI‐BP	−4.93(−8.02, −1.84)	***.002***	***.04***	−6.04(−9.89, −2.20)	***0.003***	−6.91(−11.20, −2.62)	***.002***	***.044***	−7.67(−13.03, −2.31)	***.006***
HAMD	−0.38(−0.94, 0.18)	.181				−0.54(−1.32, 0.24)	.169			
MADRS	−0.37(−0.76, 0.02)	.059				−0.49(−10.3, 0.45)	.072			
HAMA	−0.51(−1.17, 0.16)	.131				−0.82(−1.73, 0.10)	.080			
PANSS−T	−0.39(−0.77, −0.02)	***.041***				−0.64(−1.15, −0.13)	***.016***			
PANSS‐P	−1.66(−3.62, 0.31)	.097				−2.50(−5.21, 0.22)	.070			
PANSS‐N	−0.66(−1.70, 0.37)	.202				−.93(−2.37, 0.51)	.198			
PANSS‐G	−0.40(−0.90, 0.10)	.111				−0.70(−1.38, −0.02)	***.044***			
DSSS	−0.02(−0.14, 0.13)	.774				0.01(−0.19, 0.21)	.916			
DSST	−0.09(−0.25, 0.05)	.203				−0.09(−0.29, 0.11)	.360			
PDQD	−0.04(−0.48, 0.40)	.861				0.13(−0.55, 0.58)	0.963			
PCS	−0.16(−0.43, 0.11)	.239				−0.18(−0.52, 0.17)	.315			
MCS	0.11(−0.04, 0.26)	.161				0.08(−0.12, 0.27)	.455			
SDS	−0.26(−2.01, 1.49)	.765				0.20(−2.06, 2.45)	.863			

Abbreviations: BMI: body mass index; CGI‐BP: Clinical Global Impression—Bipolar Version—Severity of Illness; DBP: diastolic blood pressure; DSSS: Depression and Somatic Symptom Scale; DSST: Digit Symbol Substitution Test; FBS: fasting blood sugar; HAMA: Hamilton Anxiety Rating Scale; HAMD: 17‐item Hamilton Depression Rating Scale; HDL: high‐density lipoprotein cholesterol; MADRS: Montgomery‐Åsberg Depression Rating Scale; MCS: Mental Component Summary of the 12‐Item Short Form Health Survey; PANSS‐G: general scores in PANSS; PANSS‐N: negative scores in PANSS; PANSS‐P: positive scores in PANSS; PANSS‐T: total scores in Positive and Negative Syndrome Scale (PANSS); PCS: Physical Component Summary of the 12‐Item Short Form Health Survey; PDQD: Perceived Deficits Questionnaire‐Depression; SBP: systolic blood pressure; SDS: Sheehan Disability Scale; YMRS: Young Mania Rating Scale.

^a^ Bold italicized values represent the *p*‐value less than .05.

^b^ Only a corrected *p*‐value less than .05 after correction for multiple comparisons was listed in the table.

## 4 DISCUSSION

In this study, we explored the role of cEPCs in BPD and BPD‐related clinical presentations in patients with BPD. Although no significant difference in immature and mature cEPC counts was noted between the patients with BPD and the HCs, we discovered that the count of both types of cEPCs exhibited significant inverse correlations with YMRS, CGI‐BP, and PANSS‐P scores (Table 2). The greater strength of both manic and positive symptoms in the patients with BPD accompanied lower immature and mature cEPC counts. Furthermore, after controlling for physiological (e.g., blood pressure and BMI) and biochemical (e.g., FBS, and HDL) effects in multivariate stepwise regression, both immature and mature cEPC counts were inversely associated with the global severity of BPD (i.e., CGI‐BP; Table 3). The relationship between the cEPC count and manic symptom severity indicates that the cEPC count may be a cellular marker of the vascular–bipolar link (Goldstein et al., 2020).

Evidence has suggested that variations in the cEPC count are related to affective symptoms. Such a finding mainly originates from the effects of depressive symptom severity and depressive disorders on the cEPC count (N. Yang et al., 2021). Most findings have shown that lower cEPC numbers are associated with high depressive symptom severity and depressive disorder (N. Yang et al., 2021). However, a contradictory finding has also been reported (Liou et al., 2021; N. Yang et al., 2021), which may be explainable by variations in the study populations, the effect of antidepressants, or the duration of antidepressant treatment (Liou et al., 2021; N. Yang et al., 2021). Although studies have shown the association between depressive symptoms and depressive disorder, the relationship of the cEPC count with manic and related symptoms in BPD has not been studied. To the best of our knowledge, the present study is the first to investigate the relationship of both manic and positive BPD‐related symptoms with cEPC counts, and this study showed that stronger manic and positive symptoms are correlated with lower cEPC numbers in patients with BPD. Fiedorowicz et al. (2012) enrolled a cohort of individuals from the National Institute of Mental Health Collaborative Depression Study, and they demonstrated that participants with more severe manic symptoms exhibited poorer endothelial function, as measured by flow‐mediated dilation (FMD) of the brachial artery (Fiedorowicz et al., 2012). Because cEPCs are direct indicators of endothelial function (Chopra et al., 2018), the finding of an inverse correlation between manic symptom severity and cEPC numbers in present study is in accordance with the finding of Fiedorowicz et al. (2012), and the finding suggests that endothelial dysfunction is associated with low cEPC numbers in patients with BPD.

The reasons underlying the inverse relationship between cEPC counts and manic symptom severity in patients with BPD remain to be determined. The numbers of cEPCs could be affected by fasting, smoking, serum cholesterol, oxidized LDL levels, hypertension, or DM (Chopra et al., 2018). In the present study, we controlled for confounding factors such as being a smoker, BMI, blood pressure, FBS, and HDL by using Kendall's tau‐b partial correlation. We observed that both immature and mature cEPC counts continued to exhibit inverse correlations with YMRS and CGI‐BP scores (Table 2). Furthermore, stepwise regressions revealed that the CGI‐BP score was inversely associated with immature and mature cEPC counts (Table 3). These results suggest that the inverse relationship between cEPC counts and manic symptom severity is independent of cEPC‐related cardiovascular factors.

Numerous factors have been identified as being related to cEPC counts, such as inflammation and oxidative stress. The numbers of cEPCs are sensitive to inflammatory markers and mediators, including tumor necrosis factor‐α (TNF‐α), C‐reactive protein (CRP), and interleukin 6 (IL‐6). CRP, an acute phase marker of systemic inflammation, exerts a direct inhibitory effect on cEPC differentiation, migration, proliferation, adhesion, and angiogenic activity (Verma et al., 2004) and enhances the apoptosis of cEPCs (J. Chen et al., 2012). TNF‐α also significantly reduces proliferation, migration, adhesion, and angiogenic functions and the quantity of cEPC, and a negative correlation has been reported between the TNF‐α concentration and cEPC count (T.G. Chen et al., 2011; Seeger et al., 2005). Similarly, although IL‐6 stimulates cEPC proliferation and migration, the cEPC count has been found to exhibit an inverse correlation with the IL‐6 concentration in patients with chronic hemodialysis or first‐episode depression (Ozkok et al., 2013; L. Yang et al., 2011). Inflammatory processes also play a pivotal role in the pathophysiology of BPD (Muneer, 2016). Increasing evidence has shown high levels of circulatory inflammatory cytokines in distinct phases of BPD (Muneer, 2016), and some inflammatory cytokines and mediators are persistently activated in both symptomatic and euthymic patients with BPD (Solmi et al., 2021; Vares et al., 2020). Oxidative stress is defined as an imbalance between pro‐ and antioxidants or between the production and removal of ROS, wherein excessive pro‐oxidant activity and ROS production lead to several effects deleterious to aerobic life‐forms (Sies, 1991). Preclinical studies have demonstrated that high ROS (e.g., hydrogen peroxide) levels and ROS production are associated with low cEPC numbers (Urbich et al., 2005). At the clinical level, the conditions associated with high systemic oxidative stress are related to low cEPC numbers (Watson et al., 2008). One study reported the involvement of oxidative stress in the pathophysiology of BPD based on findings of altered oxidative stress levels in antioxidant enzymes, nitric oxide, and the products of oxidative damage to DNA/RNA, proteins, and lipids (Jimenez‐Fernandez et al., 2021). Some oxidative stress markers and antioxidants exhibit varying levels in specific BPD phases, such as UA during euthymia and thiobarbituric acid reactive substances (TBARS) during depression in BPD (Jimenez‐Fernandez et al., 2021). In summary, the inverse relationship of cEPC counts with manic symptom severity may reflect an adaption of cEPCs to inflammatory and oxidative stress in the pathophysiology of BPD.

In the present study, the cEPC count was significantly negatively correlated with the PANSS‐P score (Table 2). Psychosis and its related symptoms, such as delusion, grandiosity, hallucinatory behavior, overexcitement, and hostility, are not only prominent in schizophrenia but also in other psychiatric conditions, such as BPD (Fernandez‐Garcimartin et al., 2014). The patients with BPD and psychotic symptoms have more severe mood and anxiety symptoms; higher rates of psychiatric hospitalization, suicidality, and sexual and physical abuse; and poorer psychosocial functioning and prognosis than those patients with BPD who do not have psychotic symptoms (Shalev et al., 2020). Historically, psychosis in BPD is also associated with a high risk of CVD (Prieto et al., 2015). The inverse correlation of cEPC counts with PANSS‐P scores observed in the present study is in line with the increased risk of CVD in psychotic mania (Prieto et al., 2015) and suggests the involvement of endothelial dysfunction in the susceptibility to CVD among patients with BPD and psychotic symptoms. The aforementioned inverse correlation may also suggest that cEPCs are the cellular contributors to CVD risk factors in patients with BPD, specifically for those patients with psychotic symptoms.

The numbers of immature and mature cEPCs in patients with BPD were compared with those in the HCs in this study. However, no significant differences in the median numbers of either type of cEPC were observed between the patients with BPD and the HCs (Table 1). This result is in line with the finding of Ferensztajn‐Rochowiak et al. (2017) that the cEPC numbers in patients with BPD (who were either treated or not treated with lithium) were not significantly different from the HCs. One explanation for this failure to detect a difference in cEPC counts between the patients with BPD and the controls may be that both studies were limited by small sample sizes. For the median difference in cEPC levels between groups, our study only had statistical power of .07 and .27 for small and medium effect sizes, respectively. Similarly, our study had statistical power of .24 to .55 for very weak correlation, such as the relationship between cEPC counts and depressive symptom levels. Increasing the sample size in future studies is necessary to draw robust conclusions on the association between the cEPC count and BPD. Regarding the correlation of severe manic symptoms with low cEPC numbers noted in this study, the similar numbers of cEPCs in the BPD patients compared with the HCs may be the effects of psychotropic drugs on cEPC numbers. BPD is associated with high risks of recurrence and relapse. In clinical practice, polypharmacy, including prescriptions of antipsychotics, lithium, mood stabilizers, and antidepressants, is common applied to prevent affective recurrence and maintain remission (Yatham et al., 2018). Most antipsychotic agents used in the treatment of BPD are antagonists of dopamine D2 receptors (DRD2). In a murine model, DRD2 antagonists increased the numbers of cEPCs by eliminating the negative effects of dopamine on the migration and mobilization of cEPCs (Chakroborty et al., 2008). Antidepressants can increase cEPC numbers by enhancing the mobilization and proliferation as well as inhibiting the apoptosis of cEPCs (Zan et al., 2015). Among mood stabilizers, lithium also improves the proliferation, migration, and network formation of cEPCs by inhibiting glycogen synthase kinase 3β (Cui et al., 2015). Nevertheless, the effect of valproic acid on cEPC numbers has not been thoroughly investigated. The aforementioned evidence collectively indicates that the psychotropic drugs commonly used for BPD treatment may increase cEPC counts, thus reducing the difference in cEPC numbers between the patients with BPD and the HCs. The other explanation for the similar numbers of cEPCs in the patients with BPD relative to the HCs is that the cEPC count might be a treatment (rather than a diagnostic) marker of endothelial dysfunction in CVDs or cardiometabolic diseases comorbid with BPD. This is supported by earlier studies that have shown that lower FMD is significantly correlated with higher TG, FBS, and BMI as well as higher waist circumference and manic or hypomanic symptom burden (Fiedorowicz et al., 2012; Hatch et al., 2015) in patients with the disorder. A treatment marker would be helpful to develop, refine, and monitor a treatment network on the specific dimension of symptoms or comorbid conditions (Gill et al., 2021), such as endothelial dysfunction in patients with BPD.

Endothelial dysfunction is characterized by a proinflammatory, prothrombotic, and atherogenic state (Little et al., 2021). Endothelial dysfunction occurs in the early stage of atherosclerotic vascular damage and is a crucial factor that precedes atherogenesis and plaque formation that can lead to the development and progression of CVDs (Gimbrone & Garcia‐Cardena, 2016). Endothelial dysfunction is also associated with hypertension, obesity, and diabetes (Amarasekera et al., 2021). Because patients with BPD are considered to be at risk of hypertension, obesity, diabetes, and CVD (Goldstein et al., 2020), several studies investigating endothelial vascular dysfunction in patients with BPD have been conducted, with measures of endothelial activity and endothelial dysfunction‐related arterial stiffness, such as FMD, pulse wave velocity, augmentation index (AIx), and reactive hyperemia index (RHI) (Fiedorowicz et al., 2012; Hatch et al., 2015; Murray et al., 2012; Rybakowski et al., 2006; Sodhi et al., 2012; Tong et al., 2018). However, the findings of these studies are diverse, and drawing a conclusion from them is not possible. For instance, AIx in patients with BPD or in depressed patients with mixed mood disorder was significantly higher compared with the HCs (Rybakowski et al., 2006) or age‐based population norms (Sodhi et al., 2012). However, this difference in AIx was not replicated in another similar study (Tong et al., 2018). In addition, no significant difference in FMD and RHI was observed between patients with BPD and HCs in some of the studies (Murray et al., 2012; Tong et al., 2018). These discrepancies regarding the association of impairment in endothelial function with BPD may originate from differences in participant age range, mood state, and BPD stage or from the limited sensitivity of measures for detecting differential changes in patients with BPD compared with the HCs (Tong et al., 2018). Despite the inconsistent findings on the link between endothelial dysfunction and BPD, lower FMD was significantly correlated with higher TG, FBS, and BMI and waist circumference but lower HDL (Hatch et al., 2015). Moreover, lower FMD was associated with manic or hypomanic symptom burden, suggesting that manic or hypomanic symptoms are associated with poor endothelial function (Fiedorowicz et al., 2012). Our results of lower cEPC numbers correlating with higher manic and positive symptom levels support this point of view from a cellular level and provide another piece of evidence for the link between BPD symptom severity and endothelial dysfunction.

The present study entailed some limitations. First, our study had a cross‐sectional design, precluding causal interpretations of the association of cEPC counts with mood and positive symptoms. Additional longitudinal studies with larger samples are required to elucidate the temporal relationship among cEPC count, changes in mood‐related symptoms, and pharmacological effects throughout the course of BPD. Second, the study did not investigate the relationship among cEPC function parameters (such as adhesion, migration, tube formation, and the ability of cEPCs to form colonies), BPD diagnosis, and levels of clinical symptom severity. Third, adolescent patients, older patients, or patients undergoing any acute mood‐related episode were not enrolled in this study. The findings of inverse correlations of manic and positive symptom severities with cEPC counts were obtained from the data of patients whose total YMRS scores were less than 13 (Tohen et al., 2009), and we are unable to generalize these findings to the patients with BPD in other age groups or other phases or states of the disease. Despite these shortcomings, our study is, to the best of our knowledge, the first to investigate the relationship between cEPC counts and BPD from multiple clinical perspectives including mood‐related symptom severity, cognitive dysfunction, and function disability. Our data suggest that the numbers of cEPC may play a role in vascular homeostasis in patients with BPD. Subsequent studies are warranted to verify these observed associations.

In conclusion, the present study reported low numbers of cEPC correlated with severe manic and positive symptom severity in patients with BPD. The inverse correlation of cEPC counts and the severity of manic and positive symptoms were independent of several cardiovascular factors. Although the medians of both types of cEPCs did not significantly differ between the patients with BPD and the HCs, our results suggest that the cEPC count may be a treatment, rather than a diagnostic, marker of endothelial dysfunction in patients with BPD who are at risk of CVDs (Gill et al., 2021). Despite the exploratory and preliminary nature of the study, the inverse correlation of cEPC numbers and symptom severity implies that the cEPC count may be a cellular marker of the vascular–bipolar link (Goldstein et al., 2020) and the cellular mechanism involved in the association between BPD and CVDs.

## CONFLICT OF INTEREST

All authors have no conflict of interest and no financial support relevant to this article to disclose.

## AUTHOR CONTRIBUTIONS

Ya‐Mei Bai and Kai‐Lin Huang conceptualized and supervised the project. Ying‐Jay Liou performed data analyses and drafted the manuscript. Mu‐Hong Chen, Ju‐Wei Hsu, Kai‐Lin Huang, and Ya‐Mei Bai provided the samples and clinical data for the study. Po‐Hsun Huang designed, performed and supervised the laboratory experiments. All authors have made substantial contributions to the work, reviewed, and revised the manuscript.

## Data Availability

The data that support the findings of this study are available upon reasonable request from the corresponding author. The data are not publicly available due to privacy or ethical restrictions.

## References

[brb32570-bib-0001] Amarasekera, A. T. , Chang, D. , Schwarz, P. , & Tan, T. C. (2021). Does vascular endothelial dysfunction play a role in physical frailty and sarcopenia? A systematic review. Age and Ageing, 50(3), 725–732. 10.1093/ageing/afaa237 33951149

[brb32570-bib-0002] Anderson, A. E. , Mansolf, M. , Reise, S. P. , Savitz, A. , Salvadore, G. , Li, Q. , & Bilder, R. M. (2017). Measuring pathology using the PANSS across diagnoses: Inconsistency of the positive symptom domain across schizophrenia, schizoaffective, and bipolar disorder. Psychiatry Research, 258, 207–216. 10.1016/j.psychres.2017.08.009 28899614 PMC5681392

[brb32570-bib-0003] Carmody, T. J. , Rush, A. J. , Bernstein, I. , Warden, D. , Brannan, S. , Burnham, D. , Woo, A. , & Trivedi, M. H. (2006). The Montgomery Asberg and the Hamilton ratings of depression: A comparison of measures. European Neuropsychopharmacology, 16(8), 601–611. 10.1016/j.euroneuro.2006.04.008 16769204 PMC2151980

[brb32570-bib-0004] Carney, C. P. , & Jones, L. E. (2006). Medical comorbidity in women and men with bipolar disorders: A population‐based controlled study. Psychosomatic Medicine, 68(5), 684–691. 10.1097/01.psy.0000237316.09601.88 17012521

[brb32570-bib-0005] Chakroborty, D. , Chowdhury, U. R. , Sarkar, C. , Baral, R. , Dasgupta, P. S. , & Basu, S. (2008). Dopamine regulates endothelial progenitor cell mobilization from mouse bone marrow in tumor vascularization. Journal of Clinical Investigation, 118(4), 1380–1389. 10.1172/JCI33125 18340382 PMC2267013

[brb32570-bib-0006] Chen, J. , Jin, J. , Song, M. , Dong, H. , Zhao, G. , & Huang, L. (2012). C‐reactive protein down‐regulates endothelial nitric oxide synthase expression and promotes apoptosis in endothelial progenitor cells through receptor for advanced glycation end‐products. Gene, 496(2), 128–135. 10.1016/j.gene.2011.12.039 22301267

[brb32570-bib-0007] Chen, T. G. , Zhong, Z. Y. , Sun, G. F. , Zhou, Y. X. , & Zhao, Y. (2011). Effects of tumour necrosis factor‐alpha on activity and nitric oxide synthase of endothelial progenitor cells from peripheral blood. Cell Proliferation, 44(4), 352–359. 10.1111/j.1365-2184.2011.00764.x 21702858 PMC6496637

[brb32570-bib-0008] Chopra, H. , Hung, M. K. , Kwong, D. L. , Zhang, C. F. , & Pow, E. H. N. (2018). Insights into endothelial progenitor cells: Origin, classification, potentials, and prospects. Stem Cells International, 2018, 1. 10.1155/2018/9847015 PMC6276490 30581475

[brb32570-bib-0009] Cui, B. , Jin, J. , Ding, X. , Deng, M. , Yu, S. , Song, M. B. , Yu, Y. , Zhao, X. , Chen, J. , & Huang, L. (2015). Glycogen synthase kinase 3beta inhibition enhanced proliferation, migration and functional re‐endothelialization of endothelial progenitor cells in hypercholesterolemia microenvironment. Experimental Biology and Medicine (Maywood, N.J.), 240(12), 1752–1763. 10.1177/1535370215589908 PMC4935338 26069270

[brb32570-bib-0010] Ferensztajn‐Rochowiak, E. , Kucharska‐Mazur, J. , Samochowiec, J. , Ratajczak, M. Z. , Michalak, M. , & Rybakowski, J. K. (2017). The effect of long‐term lithium treatment of bipolar disorder on stem cells circulating in peripheral blood. The World Journal of Biological Psychiatry: The Official Journal of the World Federation of Societies of Biological Psychiatry, 18(1), 54–62. 10.3109/15622975.2016.1174301 27071327

[brb32570-bib-0011] Fernandez‐Garcimartin, H. , Bagney, A. , Moreno‐Ortega, M. , Dompablo, M. , Torio, I. , Lobo, A. , Jimenez‐Arriero, M.‐A. , Palomo, T. , & Rodriguez‐Jimenez, R. (2014). Is it possible to combine different psychotic symptom scales in bipolar disorder? Psychiatry Research, 220(3), 1090–1093. 10.1016/j.psychres.2014.10.018 25468627

[brb32570-bib-0012] Fiedorowicz, J. G. , Coryell, W. H. , Rice, J. P. , Warren, L. L. , & Haynes, W. G. (2012). Vasculopathy related to manic/hypomanic symptom burden and first‐generation antipsychotics in a sub‐sample from the collaborative depression study. Psychotherapy and Psychosomatics, 81(4), 235–243. 10.1159/000334779 22584147 PMC3567920

[brb32570-bib-0013] Gill, H. , Rosenblat, J. D. , Mansur, R. B. , & McIntyre, R. S. (2021). Inflammation and disease modification in bipolar disorders: Priority avenues for future research. Bipolar Disorders, 23(5), 442–444. 10.1111/bdi.13104 34296499

[brb32570-bib-0014] Gimbrone, M. A. Jr. , & Garcia‐Cardena, G. (2016). Endothelial cell dysfunction and the pathobiology of atherosclerosis. Circulation Research, 118(4), 620–636. 10.1161/CIRCRESAHA.115.306301 26892962 PMC4762052

[brb32570-bib-0015] Goldstein, B. I. , Baune, B. T. , Bond, D. J. , Chen, P.‐H. , Eyler, L. , Fagiolini, A. , Gomes, F. , Hajek, T. , Hatch, J. , Mcelroy, S. L. , Mcintyre, R. S. , Prieto, M. , Sylvia, L. G. , Tsai, S.‐Y. , Kcomt, A. , & Fiedorowicz, J. G. (2020). Call to action regarding the vascular‐bipolar link: A report from the Vascular Task Force of the International Society for Bipolar Disorders. Bipolar Disorders, 22(5), 440–460. 10.1111/bdi.12921 32356562 PMC7522687

[brb32570-bib-0016] Goldstein, B. I. , Schaffer, A. , Wang, S. , & Blanco, C. (2015). Excessive and premature new‐onset cardiovascular disease among adults with bipolar disorder in the US NESARC cohort. Journal of Clinical Psychiatry, 76(2), 163–169. 10.4088/JCP.14m09300 25742203

[brb32570-bib-0017] Hatch, J. , Collinger, K. , Moody, A. , Olowoyeye, O. , Zhan, J. Q. , & Goldstein, B. I. (2015). Non‐invasive vascular imaging is associated with cardiovascular risk factors among adolescents with bipolar disorder. Pediatric Cardiology, 36(1), 158–164. 10.1007/s00246-014-0980-9 25096903

[brb32570-bib-0018] Hill, J. M. , Zalos, G. , Halcox, J. P. J. , Schenke, W. H. , Waclawiw, M. A. , Quyyumi, A. A. , & Finkel, T. (2003). Circulating endothelial progenitor cells, vascular function, and cardiovascular risk. New England Journal of Medicine, 348(7), 593–600. 10.1056/NEJMoa022287 12584367

[brb32570-bib-0019] Hung, C. I. , Liu, C. Y. , Juang, Y. Y. , & Wang, S. J. (2006). The impact of migraine on patients with major depressive disorder. Headache, 46(3), 469–477. 10.1111/j.1526-4610.2006.00378.x 16618265

[brb32570-bib-0020] Jaeger, J. (2018). Digit symbol substitution test: The case for sensitivity over specificity in neuropsychological testing. Journal of Clinical Psychopharmacology, 38(5), 513–519. 10.1097/JCP.0000000000000941 30124583 PMC6291255

[brb32570-bib-0021] Jimenez‐Fernandez, S. , Gurpegui, M. , Garrote‐Rojas, D. , Gutierrez‐Rojas, L. , Carretero, M. D. , & Correll, C. U. (2021). Oxidative stress parameters and antioxidants in patients with bipolar disorder: Results from a meta‐analysis comparing patients, including stratification by polarity and euthymic status, with healthy controls. Bipolar Disorders, 23(2), 117–129. 10.1111/bdi.12980 32780547

[brb32570-bib-0022] Kay, S. R. , Fiszbein, A. , & Opler, L. A. (1987). The positive and negative syndrome scale (PANSS) for schizophrenia. Schizophrenia Bulletin, 13(2), 261–276. 10.1093/schbul/13.2.261 3616518

[brb32570-bib-0023] Lam, R. , Lamy, F.‐X. , Danchenko, N. , Yarlas, A. , White, M. K. , Rive, B. , & Saragoussi, D. (2018). Psychometric validation of the perceived deficits questionnaire‐depression (PDQ‐D) instrument in US and UK respondents with major depressive disorder. Neuropsychiatric Disease and Treatment, 14, 2861–2877. 10.2147/NDT.S175188 30464471 PMC6211374

[brb32570-bib-0024] Liou, Y. J. , Chen, M. H. , Hsu, J. W. , Huang, K. L. , Huang, P. H. , & Bai, Y. M. (2021). Associations between increased circulating endothelial progenitor cell levels and anxiety/depressive severity, cognitive deficit and function disability among patients with major depressive disorder. Science Reports, 11(1), 18221. 10.1038/s41598-021-97853-9 PMC8440504 34521977

[brb32570-bib-0025] Little, P. J. , Askew, C. D. , Xu, S. , & Kamato, D. (2021). Endothelial dysfunction and cardiovascular disease: History and analysis of the clinical utility of the relationship. Biomedicines, 9(6), 699. 10.3390/biomedicines9060699 34203043 PMC8234001

[brb32570-bib-0026] Muneer, A. (2016). Bipolar disorder: Role of inflammation and the development of disease biomarkers. Psychiatry Investigation, 13(1), 18–33. 10.4306/pi.2016.13.1.18 26766943 PMC4701682

[brb32570-bib-0027] Murray, D. P. , Metz, N. S. , Haynes, W. G. , & Fiedorowicz, J. G. (2012). Vascular function is not impaired early in the course of bipolar disorder. Journal of Psychosomatic Research, 72(3), 195–198. 10.1016/j.jpsychores.2011.12.006 22325698 PMC3278715

[brb32570-bib-0028] Ozkok, A. , Aktas, E. , Yilmaz, A. , Telci, A. , Oflaz, H. , Deniz, G. , & Yildiz, A. (2013). Decrease in endothelial progenitor cells associated with inflammation, but not with endothelial dysfunction in chronic hemodialysis patients. Clinical Nephrology, 79(1), 21–30. 10.5414/CN107318 22909781

[brb32570-bib-0029] Prieto, M. L. , Mcelroy, S. L. , Hayes, S. N. , Sutor, B. , Kung, S. , Bobo, W. V. , Fuentes, M. E. , Cuellar‐Barboza, A. B. , Crow, S. , Ösby, U. , Chauhan, M. , Westman, J. , Geske, J. R. , Colby, C. L. , Ryu, E. , Biernacka, J. M. , & Frye, M. A. (2015). Association between history of psychosis and cardiovascular disease in bipolar disorder. Bipolar Disorders, 17(5), 518–527. 10.1111/bdi.12302 26062406

[brb32570-bib-0030] Rotella, F. , Cassioli, E. , Calderani, E. , Lazzeretti, L. , Ragghianti, B. , Ricca, V. , & Mannucci, E. (2020). Long‐term metabolic and cardiovascular effects of antipsychotic drugs. A meta‐analysis of randomized controlled trials. European Neuropsychopharmacology, 32, 56–65. 10.1016/j.euroneuro.2019.12.118 31917068

[brb32570-bib-0031] Rybakowski, J. K. , Wykretowicz, A. , Heymann‐Szlachcinska, A. , & Wysocki, H. (2006). Impairment of endothelial function in unipolar and bipolar depression. Biological Psychiatry, 60(8), 889–891. 10.1016/j.biopsych.2006.03.025 16730331

[brb32570-bib-0032] Seeger, F. H. , Haendeler, J. , Walter, D. H. , Rochwalsky, U. , Reinhold, J. , Urbich, C. , RöSsig, L. , Corbaz, A. , Chvatchko, Y. , Zeiher, A. M. , & Dimmeler, S. (2005). p38 mitogen‐activated protein kinase downregulates endothelial progenitor cells. Circulation, 111(9), 1184–1191. 10.1161/01.CIR.0000157156.85397.A1 15753227

[brb32570-bib-0033] Shalev, A. , Merranko, J. , Gill, M. K. , Goldstein, T. , Liao, F. , Goldstein, B. I. , Hower, H. , Ryan, N. , Strober, M. , Iyengar, S. , Keller, M. , Yen, S. , Weinstock, L. M. , Axelson, D. , & Birmaher, B. (2020). Longitudinal course and risk factors associated with psychosis in bipolar youths. Bipolar Disorders, 22(2), 139–154. 10.1111/bdi.12877 31749297 PMC7085953

[brb32570-bib-0034] Sheehan, K. H. , & Sheehan, D. V. (2008). Assessing treatment effects in clinical trials with the Discan Metric of the Sheehan Disability Scale. International Clinical Psychopharmacology, 23(2), 70–83. 10.1097/YIC.0b013e3282f2b4d6 18301121

[brb32570-bib-0035] Sies, H. (1991). Oxidative stress: From basic research to clinical application. American Journal of Medicine, 91(3C), S31–S38. 10.1016/0002-9343(91)90281-2 1928209

[brb32570-bib-0036] Sodhi, S. K. , Linder, J. , Chenard, C. A. , del Miller , D. , Haynes, W. G. , & Fiedorowicz, J. G. (2012). Evidence for accelerated vascular aging in bipolar disorder. Journal of Psychosomatic Research, 73(3), 175–179. 10.1016/j.jpsychores.2012.06.004 22850256 PMC3410319

[brb32570-bib-0037] Solmi, M. , Suresh Sharma, M. , Osimo, E. F. , Fornaro, M. , Bortolato, B. , Croatto, G. , Miola, A. , Vieta, E. , Pariante, C. M. , Smith, L. , Fusar‐Poli, P. , Shin, J. I. , Berk, M. , & Carvalho, A. F. (2021). Peripheral levels of C‐reactive protein, tumor necrosis factor‐alpha, interleukin‐6, and interleukin‐1beta across the mood spectrum in bipolar disorder: A meta‐analysis of mean differences and variability. Brain, Behavior, and Immunity, 97, 193–203. 10.1016/j.bbi.2021.07.014 34332041

[brb32570-bib-0038] Spearing, M. K. , Post, R. M. , Leverich, G. S. , Brandt, D. , & Nolen, W. (1997). Modification of the clinical global impressions (CGI) scale for use in bipolar illness (BP): The CGI‐BP. Psychiatry Research, 73(3), 159–171. 10.1016/S0165-1781(97)00123-6 9481807

[brb32570-bib-0039] Tohen, M. , Frank, E. , Bowden, C. L. , Colom, F. , Ghaemi, S. N. , Yatham, L. N. , Malhi, G. S. , Calabrese, J. R. , Nolen, W. A. , Vieta, E. , Kapczinski, F. , Goodwin, G. M. , Suppes, T. , Sachs, G. S. , Chengappa, K. R. , Grunze, H. , Mitchell, P. B. , Kanba, S. , & Berk, M. (2009). The International Society for Bipolar Disorders (ISBD) Task Force report on the nomenclature of course and outcome in bipolar disorders. Bipolar Disorders, 11(5), 453–473. 10.1111/j.1399-5618.2009.00726.x 19624385

[brb32570-bib-0040] Tong, B. , Abosi, O. , Schmitz, S. , Myers, J. , Pierce, G. L. , & Fiedorowicz, J. G. (2018). Bipolar disorder and related mood states are not associated with endothelial function of small arteries in adults without heart disease. General Hospital Psychiatry, 51, 36–40. 10.1016/j.genhosppsych.2017.12.005 29309989 PMC5869118

[brb32570-bib-0041] Urbich, C. , Knau, A. , Fichtlscherer, S. , Walter, D. H. , Brühl, T. , Potente, M. , Hofmann, W. K. , De Vos, S. , Zeiher, A. M. , & Dimmeler, S. (2005). FOXO‐dependent expression of the proapoptotic protein Bim: Pivotal role for apoptosis signaling in endothelial progenitor cells. Faseb Journal, 19(8), 974–976. 10.1096/fj.04-2727fje 15824087

[brb32570-bib-0042] Vares, E. A. , Lehmann, S. , Sauer, C. , Pariante, C. , Wieland, F. , Soltmann, B. , Bauer, M. , & Ritter, P. (2020). Association of pro‐inflammatory cytokines with clinical features in euthymic patients with bipolar‐I‐disorder. Journal of Affective Disorders, 277, 450–455. 10.1016/j.jad.2020.07.125 32871531

[brb32570-bib-0043] Verma, S. , Kuliszewski, M. A. , Li, S.‐H. , Szmitko, P. E. , Zucco, L. , Wang, C.‐H. , Badiwala, M. V. , Mickle, D. A. G. , Weisel, R. D. , Fedak, P. W. M. , Stewart, D. J. , & Kutryk, M. J. B. (2004). C‐reactive protein attenuates endothelial progenitor cell survival, differentiation, and function: Further evidence of a mechanistic link between C‐reactive protein and cardiovascular disease. Circulation, 109(17), 2058–2067. 10.1161/01.CIR.0000127577.63323.24 15078802

[brb32570-bib-0044] Ware, J. Jr. , Kosinski, M. , & Keller, S. D. (1996). A 12‐item short‐form health survey: Construction of scales and preliminary tests of reliability and validity. Medical Care, 34(3), 220–233. 10.1097/00005650-199603000-00003 8628042

[brb32570-bib-0045] Watson, T. , Goon, P. K. , & Lip, G. Y. (2008). Endothelial progenitor cells, endothelial dysfunction, inflammation, and oxidative stress in hypertension. Antioxidants & Redox Signaling, 10(6), 1079–1088. 10.1089/ars.2007.1998 18315493

[brb32570-bib-0046] Yang, L. , Ruan, L.‐M. , Ye, H.‐H. , Cui, H.‐B. , Mu, Q.‐T. , Lou, Y.‐R. , Ji, Y.‐X. , Li, W.‐Z. , Sun, D.‐H. , & Chen, X.‐B. (2011). Depression is associated with lower circulating endothelial progenitor cells and increased inflammatory markers. Acta Neuropsychiatrica, 23(5), 235–240. 10.1111/j.1601-5215.2011.00577.x 25379895

[brb32570-bib-0047] Yang, N. , Sun, S. , Duan, G. , Lv, K. , Liang, C. , Zhang, L. , Yu, J. , Tang, Y. , & Lu, G. (2021). Advances of endothelial progenitor cells in the development of depression. Frontiers in Cellular Neuroscience, 15, 608656. 10.3389/fncel.2021.608656 34421539 PMC8375291

[brb32570-bib-0048] Yatham, L. N. , Kennedy, S. H. , Parikh, S. V. , Schaffer, A. , Bond, D. J. , Frey, B. N. , Sharma, V. , Goldstein, B. I. , Rej, S. , Beaulieu, S. , Alda, M. , Macqueen, G. , Milev, R. V. , Ravindran, A. , O'Donovan, C. , Mcintosh, D. , Lam, R. W. , Vazquez, G. , Kapczinski, F. , … Berk, M. (2018). Canadian network for mood and anxiety treatments (CANMAT) and International Society for Bipolar Disorders (ISBD) 2018 guidelines for the management of patients with bipolar disorder. Bipolar Disorders, 20(2), 97–170. 10.1111/bdi.12609 29536616 PMC5947163

[brb32570-bib-0049] Young, R. C. , Biggs, J. T. , Ziegler, V. E. , & Meyer, D. A. (1978). A rating scale for mania: Reliability, validity and sensitivity. British Journal of Psychiatry, 133, 429–435. 10.1192/bjp.133.5.429 728692

[brb32570-bib-0050] Zan, T. , Li, H. , Du, Z. , Gu, B. , Liu, K. , & Li, Q. (2015). Enhanced endothelial progenitor cell mobilization and function through direct manipulation of hypoxia inducible factor‐1alpha. Cell Biochemistry and Function, 33(3), 143–149. 10.1002/cbf.3091 25801228

